# Healed Lesions of Human Cutaneous Leishmaniasis Caused By *Leishmania major* Do Not Shelter Persistent Residual Parasites

**DOI:** 10.3389/fcimb.2022.839216

**Published:** 2022-07-27

**Authors:** Rabiaa M. Sghaier, Fouad Benhnini, Fatma Z. Guerfali, Hanène Attia, Aymen Bali, Amor Zaatour, Ghada Mkannez, Adel Gharbi, Nabil Belhaj-Hamida, Hichem Dridi, Afif Ben-Salah, Koussay Dellagi, Dhafer Laouini

**Affiliations:** ^1^LR16IPT02, Laboratory of Transmission, Control and Immunobiology of Infections (LTCII), Institut Pasteur de Tunis, Tunis-Belvédère, Tunisia; ^2^Université Tunis El Manar, Tunis, Tunisia; ^3^Service of Medical Epidemiology, Institut Pasteur de Tunis, Tunis-Belvédère, Tunisia; ^4^Department of Family and Community Medicine, College of Medicine and Medical Sciences, Arabian Gulf University, Manama, Bahrain

**Keywords:** *Leishmania major*, SCAR, parasite persistence, human cutaneous leishmaniasis, Zoonotic

## Abstract

In human cutaneous leishmaniasis (HCL) caused by *Leishmania* (*L.*) *major*, the cutaneous lesions heal spontaneously and induce a Th1-type immunity that confers solid protection against reinfection. The same holds true for the experimental leishmaniasis induced by *L. major* in C57BL/6 mice where residual parasites persist after spontaneous clinical cure and induce sustainable memory immune responses and resistance to reinfection. Whether residual parasites also persist in scars of cured HCL caused by *L. major* is still unknown. Cutaneous scars from 53 volunteers with healed HCL caused by *L. major* were biopsied and the tissue sample homogenates were analyzed for residual parasites by four methods: i) microscope detection of amastigotes, ii) parasite culture by inoculation on biphasic medium, iii) inoculation of tissue exctracts to the footpad of BALB/c mice, an inbred strain highly susceptible to *L. major*, and iv) amplification of parasite kDNA by a highly sensitive real-time PCR (RT-PCR). Our results show that the scars of healed lesions of HCL caused by *L. major* do not contain detectable residual parasites, suggesting that this form likely induces a sterile cure at least within the scars. This feature contrasts with other *Leishmania* species causing chronic, diffuse, or recidivating forms of leishmaniasis where parasites do persist in healed lesions. The possibility that alternative mechanisms to parasite persistence are needed to boost and maintain long-term immunity to *L. major*, should be taken into consideration in vaccine development against *L. major* infection.

## Introduction

Human leishmaniasis are a group of diseases caused by protozoan parasites of the genus *Leishmania* (*L.*), which are transmitted by the bite of female phlebotomine sand flies during blood meal. Human cutaneous leishmaniasis (HCL) are endemic in the Old World and in the Americas and are expressed either as self-healing sores or as more chronic or disseminated muco-cutaneous lesions depending on the causative species (Reithinger et al., 2007).

Three forms of HCL coexist in Tunisia: i) the so-called “zoonotic” HCL caused by *L. major* which is the most prevalent dermotropic species in the country causing the heaviest burden, ii) the chronic form of HCL caused by *L. killicki* (syn. *L. tropica*), and iii) the sporadic form of HCL due to *L. infantum* (Aoun and Bouratbine, 2014). HCL caused by *L. major* is a rather benign disease presenting most frequently as single or multiple crusted cutaneous ulcers developing at the site of parasite inoculation. *Leishmania* metacyclic promastigotes, once inoculated by the sand fly bite, multiply within intradermal polynuclear cells and macrophages, leading to an ulcerated lesion, which appears as the Th1 host immune responses to *Leishmania* antigens emerge. Ultimately, all lesions caused by *L. major* will spontaneously heal within few weeks or months, leaving scars more or less prominent and occasionally disfiguring when they are located on the face. Individuals, living in countries endemic for HCL due to L. major, develop after a primary infection, a stron and possibly lifelong immunity against reinfection. This feature would suggest that a vaccine against this form of HCL could be readily developed. However and despite a wealth of efforts, there is still no safe and effective vaccine (Sacks, 2014). Only leishmanization, i.e., the intentional intradermal inoculation of virulent live *L. major* parasites in hidden areas of the body of healthy people, has been shown to be largely effective against reinfection (Noazin et al., 2008). This practice was abandoned due to concerns about decreasing immunity to diphtheria, pertussis, and tetanus vaccine in children following leishmanization and the risk that inoculated parasites induce protracted non healing lesions in occasional vaccinated persons (Nadim et al., 1983; Modabber, 1995; Hosseini et al., 2005).

Seminal studies on murine experimental leishmaniasis have shown that IFN-γ produced by CD4+ and CD8+ T cells mediates cure and also accounts for the maintenance of cell-mediated immunity against *L. major* and the resistance to a second challenge (Muller, 1992; Muller et al., 1993). The induction of IFN-γ relies mainly on IL-12 produced by dendritic and other antigen-presenting cells (Constantinescu et al., 1998; Park et al., 2000). In immune mice, in addition to circulating CD4+ T cells bearing markers of effector, effector memory, and central memory subsets, each playing a role in resistance to reinfection (Colpitts and Scott, 2010), skin-resident memory T cells were also described (Glennie et al., 2015). Importantly, in murine experimental leishmaniasis, the persistence of residual parasites in host tissues appeared as a rule after apparent cure and is considered key to the maintenance on the long-term anti-*Leishmania* memory responses and resistance to reinfection (Aebischer et al., 1993; Aebischer, 1994; Stenger et al., 1996; Belkaid et al., 2002a). Conversely, a complete elimination of the parasite after cure leads to loss of resistance to a second parasite challenge (Uzonna et al., 2001).

The aim of this study is to address the issue of parasite persistence in the scars of HCL due to *L. major* after disease cure. This was done by examining biopsies of scars obtained from healthy volunteers who have had a prasitologically proven CL, 1-5 years ago, that spontaneously healed.

## Materials and Methods

### Ethical Statements

The study protocol, the informed consent forms, and the sampling/experimental procedures were reviewed and approved by the Institut Pasteur de Tunis Ethical Review Board (PV 07/07). All procedures were conducted in compliance with national guidelines for the protection of human subjects from research risks. Animal experiments were performed in compliance with the directive 86/609/EEC of the European parliament and the council on the protection of animals used for scientific purposes, in agreement with the guidelines of International Guiding Principles for Biomedical Research Involving Animals and with the approval of the Ethical Review Board of Institut Pasteur de Tunis (PV 07/07).

### Study Area and Patients

A total of 53 individuals were included in this study. They were living in the governorates of Kairouan and Sidi Bouzid, two areas in Central Tunisia that share the same topography and climate and are both endemic for HCL caused by *L. major*. Inclusion criteria in the study were as follows: i) healthy individuals, men or women, 18 to 50 years old, ii) presence of scars on the skin, characteristic of healed lesions of HCL due to *L. major*, iii) past history of proven cutaneous leishmaniasis based on medical records and parasite detection by direct smear or culture and formal identification of parasite species as *L. major*, and iv) giving their informed written consent for having one scar located on the limbs or trunk punch biopsied. Exclusion criteria were as follows: all individuals with a ZCL history who i) have scars located only on the face or on sensitive areas of the body such as fingers and near the joints, ii) have a medical record of chronic diseases such as tuberculosis, hypertension, diabetes, or hemophilic patients, and iii) are pregnant or breast-feeding women. As a negative control, a skin biopsy was obtained after written consent from a healthy 35-year old women presenting no dermal disease and living in an area free of HCL. All biopsies were sampled during February and March of year 2010, before the transmission season (which occurs between May and September), to avoid any false positive result due to a recent inoculation of parasites by an infected sand fly.

### Sample Collection and Study Design

A single punch biopsy was performed on each volunteer with past history of HCL included in the study, irrespective of the number of scars detected on his body. In case of multiple scars, the sampling was done on a scar located on the upper left side. Punch biopsies (2-3 mm) were taken under sterile conditions with a single-use puncher, after local anesthesia with xylocaine containing 1% adrenalin, using a sterile single-use puncher. The biopsy was taken at the margin of the scar, near the normal skin. The biopsy site was protected with gauze after sampling, and the volunteer was asked to remain in the health center under surveillance for at least 30 min. All individuals were reexamined 1 month later to assess the healing of the biopsy site and to receive an ointment (Cicatryl^®^) to speed the scaring process and to improve the scar quality. A third visit, 3 months after enrolment, allowed to certify final healing and absence of adverse events.

### Sample Processing

The exudate extruded from each punch biopsy was smeared on two glass slides, air dried and fixed in methanol. Each biopsy was then immediately and aseptically triturated in 600 µl of Schneider medium supplemented with antibiotics and glutamine and grinded in a Dounce homogenizer. The homogenate was divided into three equal parts. One part was inoculated into two tubes of biphasic NNN culture medium; and one part was inoculated subcutaneously into two footpads of a 4- to 6-week-old female BALB/c mouse (Janvier Company, Le Genest-Saint-Isle, France); the third part of the homogenate was snap frozen in liquid nitrogen for subsequent DNA extraction and parasite quantification by real-time PCR.

### Sample Analysis

**Figure 1:**
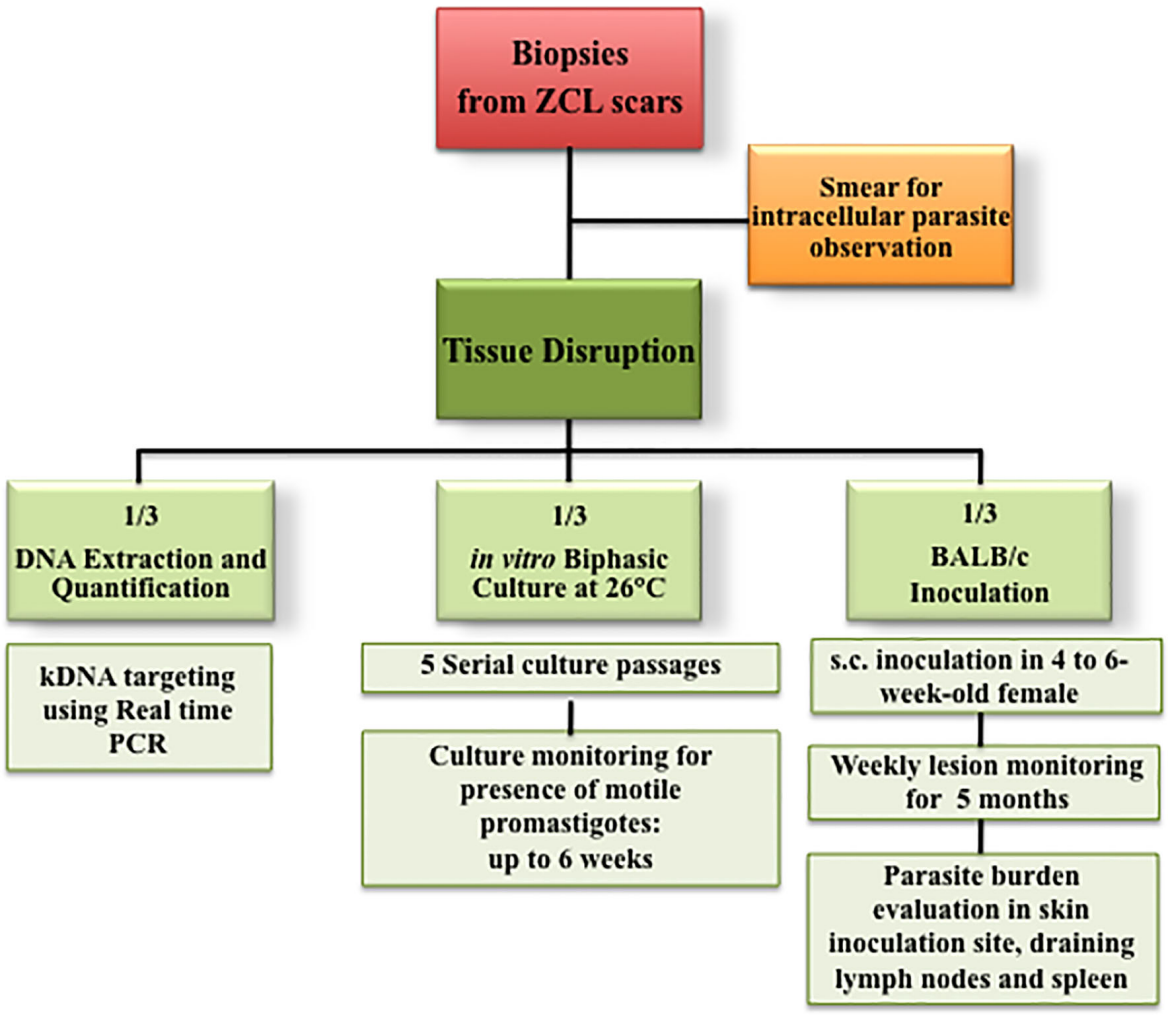
Workflow chart of the experimental design for parasite detection in zoonotic Human cutaneous leishmaniasis-healed scars.

**Figure 1** shows the workflow followed in this study. The biopsy exudates smeared on slides were Giemsa stained and examined under light microscopy for detection of intracellular amastigotes. Biopsy homogenates inoculated into biphasic media were incubated at 26°C for 5–6 days then carefully checked under a microscope. Aliquots were systematically passaged five times to a new fresh medium at weekly intervals and repeatedly checked for parasite growth for at least 6 weeks. The BALB/c mice, which were inoculated with the biopsy homogenates, were followed up at weekly intervals for 5 months and then killed. Three tissues were systematically collected from each mice (the skin at the inoculation site, the lymph node draining the inoculation site and the spleen), homogenized and then inoculated into a biphasic culture medium and checked for parasite presence as described above. A fraction of each homogenate was also DNA extracted for real-time PCR detection of *L. major* DNA. The parasite load in inoculated mice was also determined by limiting-dilution method following a protocol described elsewhere (Almeida et al., 1993; Benhnini et al., 2009). After 7–10 days of culture at 26°C, parasites were checked microscopically.

### DNA Extraction

DNA was isolated from tissue homogenates using the DNeasy Tissue Kit (Qiagen, Hilden, Germany) in accordance with the manufacturer’s protocol. Tissue homogenates were digested with proteinase K during 3 h at 56°C under agitation and then treated with RNase A. DNA was eluted in the TAE buffer, quantified using NanoDrop 1000, and stored at -20°C until use. The same protocol was followed to extract DNA from three different L. major strains (TMRC8, TMRC22 and TMRC24) using 10^8^ parasites per extraction. These DNA allowed to setting up the PCR quantification curves and check their reproducibility.

### Parasite Quantification by Real-Time PCR

*L. major* promastigotes of the GLC94 strain (MHOM/TN/95/GLC94) were grown at 26°C in RPMI 1640 medium supplemented with 10% heat-inactivated fetal calf serum, 100 U of penicillin, and 100 mg of streptomycin/ml. The parasites were harvested by centrifugation and washed twice with phosphate-buffered saline. DNA was extracted from 10^8^ parasites (counted using a Neubauer hemocytometer) following the protocol described above.

For accurate quantification of parasites, and after testing several published protocols, the TaqMan-based real-time PCR described by Mary et al. was selected for the following steps (Mary et al., 2004). This method amplifies a 116-bp DNA sequence in a kinetoplast mini-circle (kDNA), a structure present at about 10,000 copies per single parasite (Mary et al., 2004).

A standard curve was constructed using 10-fold serially diluted GLC94 *L. major* DNA from 1 ng to 0.01 fg per reaction. An Applied Biosystems qPCR master mix (#600549-51), 300 nM of forward and reverse primers, and 100 nM of TaqMan probe, as described elsewhere, were used (Mary et al., 2004). Assays were performed in 25 µl final volume and run three times in triplicate for each run on an ABI Prism 7500HT sequence detection system (Applied Biosystems, Foster City, CA, USA). For analysis, 7500 Software (v2.0.1) was used. A threshold cycle value (Ct) was calculated for each sample by determining the point at which the fluorescence exceeded the threshold limit. A standard curve was obtained by plotting the Ct values against each standard of known concentration parasite DNA. Standard curves were also constructed using intracellular amastigote-stage parasites purified by Percoll gradient from tissues of BALB/c mice previously infected with GLC94 *L. major* promastigotes.

In addition, 10-fold serially diluted GLC 94 DNA parasites (from 1 ng to 0.01 fg per reaction) were mixed with (i) purified PBMC cells (10^6^ cells), (ii) human skin tissue obtained from a healthy volunteer living in a non-endemic area of ZCL and with no leishmaniasis history, or (iii) murine skin tissue.

In order to mimic the method used to quantify intact parasites in the extacts of biopsies of scars from human volunteers with past cured HCL, tissue extracts from, (i) PBMC (10^6^ cells) obtained from the blood of a healthy volunteer subject, (ii) a skin biopsy from a healthy human volunteer, and (iii) a mouse skin biopsy. These tissues extracts were spiked with serial dilutions of purified *L. major* parasites (strain GLC94) starting from 10^7^ to 0.1 parasites with 10-fold decreasing numbers. The mixes were extracted for DNA, and amplified using kDNA-specific primers. Non spiked extracts were used as negative controls for these experiments. To detect the presence of putative PCR inhibitors in the tissue extracts, two endogeneous genes were used as positive control and tested by PCR amplification: i) the human albumin gene (human-albumin-forward 5′-GCTGTCATCTCTTGTGGGCTGT-3′ [100 nmol]; human-Albumin-Reverse 5′-ACTCATGGGAGCTGCTGGTTC-3′ [100 nmol] and human-Albumin probe 5′-GGAGAGATTTGTGTGGGCATGACA-3’ [100 nmol]) and ii) the single-copy-number mouse brain-derived neurotrophic factor (BDNF) gene (GenBank accession number NM007540; murine-BDN-Forward 5′-TTGGATGCCGCAAACATGTC-3′ [300 nmol]; murine-BDNF-Reverse 5′CTGCCGCTGTGACC CACTC-3′ [300 nmol] murine-BDNF probe 5′-TCACACACGCTCAGCTCCCCACGG-3′ [200 nmol]) (Laurendeau et al., 1999; Bretagne et al., 2001).

DNA extraction (10 ng per experiment) and RT-PCR amplification were conducted on each human scar biopsy or on tissues (skin, draining lymph node, and spleen, see above) sampled from the BALB/c mice that have been inoculated with biopsy homogenates obtained from HCL scars. All experiments were done in triplicates following the same PCR amplification protocol to detect kinetoplastic parasite DNA.

## Results

Following a workflow chart described in **Figure 1**, each collected biopsy has been (i) smeared on glass slides and observed, (ii) inoculated into a biphasic NNN culture medium, and (ii) inoculated subcutaneously into footpads of BALB/c mouse, and (iv) DNA was extracted for parasite quantification by real-time PCR.

**Figure 2:**
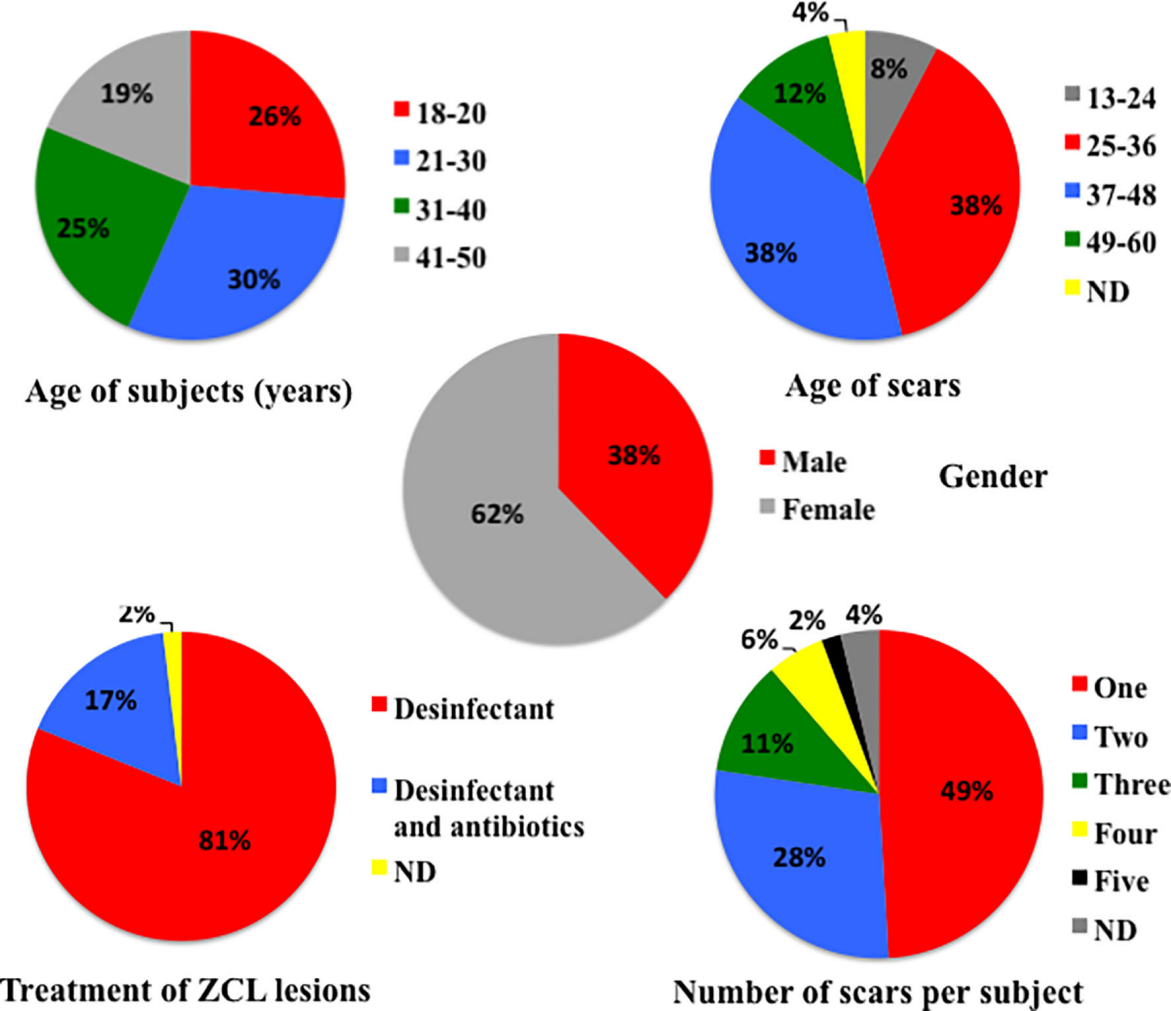
Age, gender, and number of scars of healed-patient distribution; age and localization of scars distribution; and nature of treatment taken during the zoonotic cutaneous leishmaniasis by the 53 patients included in this study and from whom biopsies of scars were obtained.

Fifty-three volunteers, bearing typical scars of healed HCL lesions following a parasitologically proven *L. major* infection, were included in the study. **Table 1** and **Figure 2** show the characteristics of each subject. They were 18–50 years old (mean 29.7 years), and 62% were women. The age range of the biopsied scars was 1 to 5 years, with most scars (76%) being aged 25 to 48 months. Most subjects had one or two scars on their body (26 and 12 individuals, respectively). The scars that were biopsied were located on the chest, the upper limbs, and the lower limbs in 1, 20, and 32 cases, respectively (**Table 1**). With regard to the original HCL, the active lesions in all volunteers have healed spontaneously: no Glucantime had been used in any patient, and only disinfection was locally applied in all cases, with topical antibiotics in nine cases.

**Table 1:** Demographic characteristics and disease history of 53 healthy volunteers clinically cured of Human Cutaneous Leishmaniasis due to *L. major* who are included in the present study.

Patient #	Age (years)	Gender	Last zoonotic HCL episode	Scar number	Treatment after infection^1^		Scar age (months)	Biopsied scar location	
					D^2^	A^3^			
4	20	F	2006	1	Y	Y	ND		Calf
6	41	M	2006	1	Y	N	36		Forearm
7	38	F	2007	1	Y	N	30		Forearm
9	20	F	2006	4	Y	N	36		Forearm
12	40	F	2005	2	Y	N	48		Upper arm
13	46	M	2006	1	Y	N	36		Calf
16	18	F	2007	4	Y	Y	29		Upper arm
17	19	M	2006	1	Y	N	42		Shin
18	23	M	2006	1	Y	Y	41		Foot
22	48	M	2006	3	Y	N	40		Forearm
23	21	F	2007	1	Y	N	ND		Forearm
25	47	F	2006	1	Y	N	41		Shin
26	35	F	2007	2	Y	N	30		Calf
27	49	F	2006	2	Y	N	41		Forearm
28	39	F	2007	2	Y	N	29		Thigh
29	18	M	2008	2	Y	N	18		Upper arm
31	26	F	ND	ND	Y	N	ND		Foot
32	19	F	2005	2	Y	N	41		Shin
33	36	F	2006	5	Y	Y	41		Upper arm
34	41	M	2006	3	Y	N	36		Chest
35	19	F	2007	1	Y	N	29		Upper arm
37	19	M	2007	1	Y	N	30		Foot
39	18	F	2007	1	Y	N	29		Thigh
41	48	M	2006	2	Y	N	29		Foot
42	31	M	2005	1	Y	N	42		Shin
43	47	F	2005	3	Y	N	42		Foot
44	19	F	2004	ND	Y	ND	54		Foot
45	23	F	2006	2	Y	N	30		Foot
46	26	M	2005	1	Y	N	42		Forearm
47	24	F	2006	1	Y	N	30		Foot
48	19	F	2007	1	Y	N	18		Calf
50	24	F	2006	3	Y	N	30		Shin
51	44	F	2006	1	Y	N	30		Shin
53	24	M	2007	2	Y	N	18		Upper arm
54	24	F	2005	3	Y	N	41		Upper arm
55	19	M	2005	1	Y	N	38		Upper arm
57	21	M	2005	1	Y	N	36		Shin
58	21	M	2007	1	Y	N	18		Upper arm
59	18	F	2004	2	Y	N	54		Foot
60	27	F	ND	2	Y	Y	42		Thigh
61	35	M	2006	4	Y	N	29		Foot
62	34	M	2005	3	Y	N	42		Shin
64	38	F	2005	2	Y	N	53		Shin
65	37	F	2005	2	Y	N	42		Shin
66	26	F	2006	1	Y	N	30		Forearm
67	39	M	2005	1	Y	Y	53		Shin
68	24	F	2005	2	Y	Y	38		Shin
69	19	M	2005	1	Y	Y	41		Calf
70	36	F	2005	2	Y	N	38		Shin
71	25	F	2006	1	Y	Y	29		Forearm
72	24	M	2004	1	Y	N	54		Forearm
73	45	F	2004	1	Y	N	50		Calf
74	36	F	2005	1	Y	N	42		Forearm

^1^Treatment after infection during the last zoonotic HCL episode.

^2^ Local disinfection.

^3^ Local antibiotics.

### Sensitivity and Reproducibility of PCR Assays

To determine the sensitivity and reproducibility of the PCR assay, a known amount of parasite DNA extracted from promastigotes and amastigotes of four *L. major* strains was serially diluted and PCR amplified. This allowed to detect 10^-6^ ng DNA with a Ct <35 from both promastigote and amastigote forms, which approximately correspond to 0.001 parasite per PCR reaction (see **Supplementary Figure 1A**). The mean standard curve was linear with a correlation coefficient (*R*^2^) of 0.99 and 0.97 for both promastigote and amastigote curves, respectively (see **Supplementary Figure 1A**).

We then conducted similar experiments in which serially diluted DNA parasite extracted from either promastigote or amastigote stages was mixed with tissue homogenates of either human (PBMC), or normal human or murine skin biopsies. A similar sensitivity was obtained with a correlation coefficient (*R*^2^) of around 0.95, allowing the detection of the equivalent of about 0.001 parasite per PCR reaction in different samples. All curves showed similar slopes (see **Supplementary Figures 1B, C**). Finally, serially diluted amounts of intact parasites (at either amastigote or promastigote stage) were mixed with different fixed amounts of tissue homogenates (human PBMC, human, and murine skin tissues), then extracted for DNA and specifically amplified for kDNA primers. We were able to detect the presence of about one parasite per sample normalized in reference to human or mouse housekeeping genes (data not shown). PCR experiments performed on a skin biopsy obtained from a volunteer living in an area free of HCL and denying any leishmaniasis history and from non-infected BALB/c mice, respectively, yielded constantly negative results for parasite detection.

All these results indicate that the number of mini-circles (corresponding to kDNA quantities in each parasite) harbored by Tunisian *L. major* strains and by the two parasite stages was stable and that kDNA can be detected at a very low level (10^-6^ ng DNA corresponding to 0.001 parasite or one intact mixed parasite per sample). Hence, we considered that the method was sensitive enough in our quest to detect residual parasite in scars of HCL due to *L. major*.

### Parasite Detection in HCL Scars

All our attempts to detect any residual *Leishmania* parasite in the biopsies of HCL scars using several experimental approaches yielded essentially negative results. Firstly, no amastigote could be detected under light microscopy in any serosity extruded from biopsy material and smeared on slides. Secondly, *in vitro* culture of homogenates of scar biopsies revealed no growth of any detectable parasites in all samples after up to five passages and a 6-week monitoring.

Thirdly, mice inoculated in the footpad with homogenates of the biopsies of human scars did not show any clinical sign suggestive of infection, up to 5 months after injection. Fourthly, biopsies collected on these apparently asymptomatic mice from the inoculation site, from a lymph node draining the inoculation site, and from the spleen were all negative for parasite growth after inoculation to a biphasic culture medium and for parasite kDNA detection by real-time PCR. Finally, the use of this highly sensitive PCR assay showed no amplification of kDNA in the DNA extracted from biopsies of human scars despite the high sensitivity of the method. These negative results were observed irrespective to the scar localization (chest, upper and lower limbs), to the scar age (from 1 year to more than 5 years), and to the age of the subject from whom the biopsies were obtained (18- to 50 year-old).

## Discussion

The research question addressed in this work is whether the immunity acquired after the spontaneous cure of HCL caused by *L. major* is sterilizing or not, a question that has not been addressed so far for this parasite species. Practically, we tested whether the healed cutaneous lesions were totally cleared of the parasite or if residual parasites persist in the scars within resident cells.

Based on multiple experimental approaches targeting a large series of cured human HCL due to *L. major*, we did not get any evidence that parasites persist at a detectable level within the cutaneous scars (i.e. the site of parasite inoculation). Four techniques were used: direct microscopic observation of amastigotes, culture on biphasic medium, inoculation to BALB/c and amplification of parasite kDNA by a highly sensitive PCR. In similar studies conducted on American forms of HCL, these techniques have proved to be enough sensitive as to readily detect persistent parasites (Schubach et al., 1998; Mendonca et al., 2004). Although we cannot exclude that residual parasites might be located elsewhere in the body of the volunteers (for instance in the lymph nodes draining the lesion site), our results support the idea that this form of cutaneous leishmaniasis, in contrast to others (see hereafter), may lead to complete elimination of the parasites from the healed lesions. This conclusion is in keeping with our current experience in Tunisia where thousands of HCL cases caused by *L major* have been clinically evaluated so far we could not document any case of lesion reactivation at the site of a healed scar. HCL recurrence may be occasionally observed in rare patients, but the lesions were regularly located in new anatomic sites and likely represent reinfection after new contaminating bites. Unfortunately no parasite sequencing data are available to ascertain this conclusion. In contrast to our negative results in HCL due to *L. major*, persistent *Leishmania* have been previously detected in scars of patients cured from American cutaneous leishmaniasis due to *L. braziliensis* (Saravia et al., 1990; Schubach et al., 1998; Mendonca et al., 2004). One study showed that parasite DNA could be detected in scars of up to 93% of studied subjects and parasites could be grown by culture of up to 10% *Leishmania*-positive PCR cases (Mendonca et al., 2004). A recent study suggested that mucosal sites could be a privileged niche for parasite persistence after drug treatment in HCL caused by *L*. *Viannia*, supporting the silent persistence of *Leishmania* in these anatomical sites (Martinez-Valencia et al., 2017).

Leishmaniasis recidiva cutis (LRC), apart from the rare cases described in the Americas (Oliveira-Neto et al., 1998), was always linked to *L. tropica* species in the Old World and never to *L. major* infection. A study conducted, on 22,838 children in Southeast Iran, a country endemic for HCL caused by different species including *L. major*, showed that parasites isolated from all LRC-reported cases belonged to *L. tropica* species (Sharifi et al., 2010), but none of the cases was due to *L. major*. Another form of leishmaniasis which is parasite persistence-dependent disease is the post-kala-azar dermal leishmaniasis (PKDL), a late complication of visceral leishmaniasis (VL). PKDL most frequently occurs after an episode of VL caused by *L. donovani* or *L. infantum* where dermal lesions appear in cured VL as consequence of skin inflammation triggered and maintained by interferon-gamma-producing immune T cells reacting with residual parasites persisting within the skin (Zijlstra et al., 2003).

Reactivation of dormant cutaneous *Leishmania* parasites after surgery or trauma or in immune-compromised individuals was previously described, except for HCL due to *L. major* (Crowe et al., 2014), and several *Leishmania* species have been found in HIV-1-infected individuals (Alvar et al., 2008). Besides viscerotropic *L. infantum* and *L. donovani*, several dermotropic variants like *L. infantum* or *L. braziliensis* have been isolated in the context of HIV-induced immune deficiency (Cruz et al., 2006; Okwor and Uzonna, 2013). With regard to *L. major* infection in the context of HIV infection, all reported cases in the literature indicate, as far as we know, that HCL occured secondarly to HIV infection. Hence, they may either represent a *de novo* cutaneous leishmaniasis caused by *L. major* inoculated by a sand fly bite to a HIV patient or alternatively indicate the reactivation by the HIV induced immunosuppression of residual dormant parasites that persisted in the scar of a previous healed HCL due to *L. major*. The distinction between the two possibilities in the context of HIV infection remains an open question (Guiguemde et al., 2003; Foulet et al., 2006; Ngouateu et al., 2015).

Our results, as well as the reports cited above, support the view that *L. major* parasites behave in infected humans in a very different way than other *Leishmania* species. In fact, no *L. major* do survive as residual parasites within the scars of cured HCL. They might not be able to survive under the pressure of the parasite specific immune responses of the host that likely activate potent sterilizing leishmanicidal activities. The impact of such conclusion on the understanding of the long-term immune responses that humans develop against *L. major* after cure of HCL, must be discussed under the light of data generated by experimental infection in mice.

In the murine experimental model of leishmaniasis, a dogma stresses the importance of parasite persistence after cure to support a sustained long-term specific memory and protection against a second challenge (Uzonna et al., 2001; Belkaid et al., 2002a; Scott, 2020). In C57BL/6 mice, an inbred strain able to control the lesion induced by *L. major* inoculation in the footpad, viable parasites were detected, at the inoculation site long after apparent clinical recovery (Stenger et al., 1996; Belkaid et al., 2002a), as well as in lymph nodes and occasionally in spleens and bone marrow (Aebischer et al., 1993; Stenger et al., 1996). Interestingly, C57BL/6 mice once recovered from a primary challenge, resist a secondary challenge as is the case in HCL due to *L. major.* In the genetically susceptible BALB/c mice, which develop a protracted and ultimately fatal disease upon challenge with *L. major*, the parasites were detected in all organs; however, mice infected with a low dose of *L. major* develop subclinical infection and the parasites were detected in their lymph nodes and footpads but not in spleen and bone marrow, whereas in asymptomatic mice, parasites were detected only in lymph nodes (Uzonna et al., 2001). These mice resist to reinfection. Conversely, a complete parasite clearance leads to loss of resistance to reinfection (Uzonna et al., 2001). These data in the mouse model of leishmaniasis show that persistence of residual parasites might be key in maintaining resistance to reinfection. CD4+CD25+ regulatory T cells, might play a central role in such phenotype by supppressing the CD4+CD25- effector anti-*Leishmania* T cells, hence avoiding complete elimination of residual parasites. A small number of parasites persisting after cure continuously stimulates T effector cells and contributes to the maintenance of long-term immunity to *L. major*, even in the absence of true memory cells (Belkaid et al., 2002b). The essentiality of parasite persistence for sustained anti-*Leishmania* immune memory was challenged by several studies in C57BL/6 and BALB/c mice, respectively (Seder and Sacks, 2004; Zaph et al., 2004).

Using adoptive transfer of polyclonal T cells from immune mice, Zaph and collaborators demonstrated that *Leishmania*-specific T central memory cells are maintained and protect mice against challenge infection, even after sterile cure and complete elimination of parasites (Seder and Sacks, 2004; Zaph et al., 2004). Indeed, in the absence of residual parasites, long-lived pathogen-independent CD4+ central memory T cells become tissue-homing effector cells upon a secondary infection and mediate protection through effector memory T cells (Seder and Sacks, 2004; Zaph et al., 2004).

Hence, central memory CD4^+^ T cells (CD62L^hi^ phenotype) can be generated upon vaccination with attenuated *Leishmania* parasites, maintained in the absence of persistent parasites, and more importantly are able to confer protection upon transfer to naïve animals (Seder and Sacks, 2004; Zaph et al., 2004). *L. major* phosphor-manno-mutase-deficient (ΔPMM) parasites, which are avirulent and non-persistent *in vivo*, but viable *in vitro*, have been used to vaccinate susceptible BALB/c mice (Kedzierski et al., 2008). Both studies strongly suggest that immunization with non-persistent parasites generate high memory precursors that can be recruited to the draining lymph nodes after a secondary exposure to antigen, and hence that parasite persistence is not required to maintain protection against reinfection.

In contrast to the *L. major* murine model, anti-parasite drug therapy in the *T. cruzi* model may completely eliminate the parasite and renders mice resistant to reinfection. This indicates that memory T cells may contribute to concomitant immunity regardless of the persistence of residual parasites (Bustamante et al., 2008; Rosenberg et al., 2016).

That parasites apparently do not persist in lesions after cure of HCL caused by *L. major* may indicate that alternative mechanisms of immunity/resistance could operate in this form of cutaneous leishmaniasis. We have previously shown that resistance to reinfection after a first episode of HCL is neither lifelong nor absolute. In addition, asymtomatic *L. major* infection constitutes a relatively frequent mode of natural immunization, especially in areas of low transmission pressure (Ben Salah et al., 2005). Indeed, when infecting parasites and/or sand fly bites are scarce, the infection in Humans is typically asymptomatic, and a higher proportion of individuals mount a Th1 immune response and revert their leishmanin skin test (LST) to positive without developing overt any detectable cutaneous sore. This leads to a global low incidence of HCL in the region (Ben Salah et al., 2005). Moreover, in a prospetive observational study of HCL due to *L. major* in an area endemic for the disease in Tunisia, of the 1,114 individuals with personal past history of HCL who were tested LST+ at enrolment, 61 (5.7%) reverted their LST to negative after one epidemiologic season, indicating that this surrogate marker of Th1 responses is not a lifelong feature (Bettaieb et al., 2020).

Hence, in endemic regions, it may turn that the continuous exposure of humans to infected sand flies lead to repetitive inoculation of very low number of parasites which boost anti-*Leishmania* immunity and maintain resistance to reinfection as long as individuals live in the area. In other parasitic diseases, i.e., malaria (Taylor-Robinson, 1998), individuals migrating from endemic regions to malaria-free countries for long periods loose their premonition state and could be reinfected upon returning to their native country, suggesting a vanishing immunity in the absence of boosting bites. Similar observation for migrants moving from *L. major*-endemic countries to *L. major*-free countries is still missing. Epidemiologic studies are warranted to clarify this issue as they will allow evaluating the duration of the protection conferred by a primary lesion to a secondary *L. major* challenge in the absence of continuous exposure to sand fly bites.

In addition to sand fly bites inoculating few parasites that boost the host immune responses, several studies have shown that inoculation of salivary gland antigens by the female phlebotomine sand fly vector during blood meal can drastically affect the immune response to the parasite and the development of disease (Gomes and Oliveira, 2012). Indeed, individuals exposed to *Lutzomyia* (*Lu*.) *longipalpis* bites displayed a strong cellular immune response to this vector’s salivary glands characterized by IFN-γ production and a DTH response at the bite site (Vinhas et al., 2007). In addition, the incidence of positive DTH to *Leishmania* antigen is higher among residents with anti-SGS IgG (Aquino et al., 2010), suggesting that immunity to *Lu. longipalpis* sand-fly saliva might be considered as a surrogate marker of protection against *L. infantum chagasi* infection (Aquino et al., 2010). Several other results suggest that a repeated exposure to *Lu. intermedia* or *Phlebotomus* (*P.*) *duboscqi* salivary gland antigens induces the expression of potentially host-protective IFN-inducible genes (Weinkopff et al., 2014), indicating that individuals presenting a Th1-polarized response to salivary antigens would be protected against CL (Reed et al., 2016). Another mechanism that might create a Th1 environment unfavorable to *L. major* reinfection in HCL-endemic regions, regardless of parasite persistence or reinfection, is the trained innate immunity (van der Meer et al., 2015). This process is operative when macrophages are activated as a consequence of continuous exposure of the host to bites of *P. papatasi*, the vector of the *L. major* parasite.

In conclusion, our results show that parasites do not persist in scars of cured HCL caused by *L. major*. This observation, if extended to other anatomic sites (e.g., lymph nodes), would indicate that this form of leishmaniasis once healed, lead to a sterile cure and hence that other mechanisms than persistent residual parasites, might be acting to sustain immunity against reinfection.

All these features are important to take into consideration in the design of vaccine. Developing new strategies to generate T memory cells to *L. major* not dependent on residual parasites is an important step in the quest for a successful anti-*L. major* vaccine (Scott, 2020).

A prospective study designed to interrogate the dynamics of potential parasite clearance, the T cell anti-*Leishmania* immune memory, and possible reactivation of disease is needed to establish the full picture of the immunological status of healed HCL and the epidemiological impact of *L. major* persistence/clearance.

## Data Availability Statement

The original contributions presented in the study are included in the article/**Supplementary Material**. Further inquiries can be directed to the corresponding author.

## Ethics Statement

The studies involving human participants were reviewed and approved by Comité d’éthique Bio-Médicale, Institut Pasteur de Tunis. The patients/participants provided their written informed consent to participate in this study. The animal study was reviewed and approved by the Comité d’éthique Bio-Médicale, Institut Pasteur de Tunis.

## Author Contributions

AB-S, KD, and DL conceived and designed the study. RS, FB, and DL prepared the research protocol with input from all authors. FBH, AZ, AG, HD, NBH, DL and ABS selected the donors and collected the human samples. RMS, FBH, FZG, HA, GM and AB performed the experiments. RMS, DL and KD wrote the first version of the manuscript. All authors interpreted results, critically reviewed the paper and contributed to the final report.

## Funding

This work was supported by an NIH/NIAID/DMID Grant Number 5P50AI074178 and the Tunisian Ministry of Higher Education and Research for AB-S, KD, and DL. The funders had no role in study design, data collection and analysis, decision to publish, or preparation of the manuscript.

## Conflict of Interest

The authors declare that the research was conducted in the absence of any commercial or financial relationships that could be construed as a potential conflict of interest.

## Publisher’s Note

All claims expressed in this article are solely those of the authors and do not necessarily represent those of their affiliated organizations, or those of the publisher, the editors and the reviewers. Any product that may be evaluated in this article, or claim that may be made by its manufacturer, is not guaranteed or endorsed by the publisher.

## Supplementary Material

Supplementary Figure 1 Linearity assessment and limit of detection of the *L. major* kDNA as a function of the number of ng of parasitic purified DNA panel **(A)**, the number of added promastigotes panel **(B)** or amastigotes panel **(C)** parasite forms to human PBMC, shuman skin or mouse skin. Means of Ct (triplicate).
