# Supplementary figures and images for: Healed Lesions of Human Cutaneous Leishmaniasis Caused By Leishmania major Do Not Shelter Persistent Residual Parasites
Source: Front Cell Infect Microbiol. 2022 Jul 27;12:839216. doi: 10.3389/fcimb.2022.839216 (PMC9363604; doi:10.3389/fcimb.2022.839216)

## Slide 1
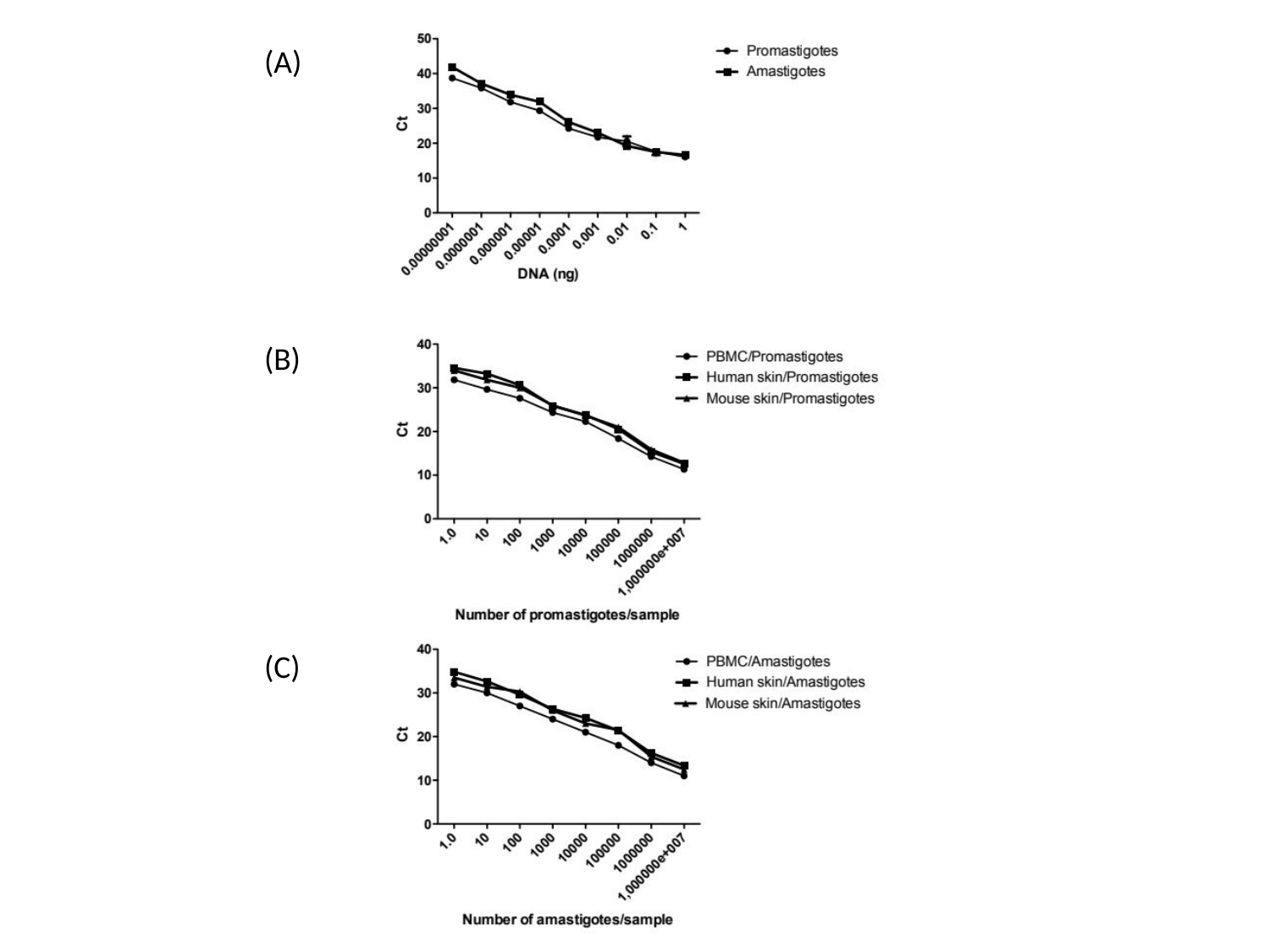

(A)
(B)
(C)

Supplement: Supplementary Figure 1 — Linearity assessment and limit of detection of the L. major kDNA as a function of the number of ng of parasitic purified DNA panel (A), the number of added promastigotes panel (B) or amastigotes panel (C) parasite forms to human PBMC, shuman skin or mouse skin. Means of Ct (triplicate). [file Presentation_1.pptx]
